# Electrophysiological Markers of Within-Network Connectivity in Major Depression

**DOI:** 10.64898/2026.08.04.26359708

**Published:** 2026-08-06

**Authors:** Yoojin Lee, Elizabeth D. Ballard, Jeffrey D. Stout, Allison Nugent, Hiroe Hu, Kelly T. Hurst, Amy Xu, Carlos A. Zarate, Jessica R. Gilbert

## Abstract

Depression and treatment-resistant depression (TRD) are significant public health issues, but the associated network-level neurobiological mechanisms remain poorly understood. This study used magnetoencephalography (MEG) to identify altered resting-state connectivity within the default mode (DMN), executive control (ECN), salience (SN), dorsal attention (DAN), motor (MN), and visual (VN) networks as potential biomarkers of depression and treatment resistance. The study recruited 168 participants (80 healthy volunteers (HVs) and 88 currently experiencing a major depressive episode (74 with TRD and 14 without TRD (noTRD))). Data Integration Analysis for Biomarker Discovery using Latent Variable Approaches for Omics Studies (DIABLO) was used to differentiate the depression, TRD, and HV subgroups and identify neural markers of depression and treatment resistance. For differentiating the depression and HV groups, the triple network model (area under the receiver operating curve (AUROC): 0.759- 0.787)—which includes the DMN, ECN, and SN—outperformed the six-network model (AUROC: 0.747-0.762) across different bandwidths. For differentiating the TRD and HV groups, the triple network model demonstrated reasonable prediction across different bandwidths (AUROC: 0.737-0.807); potential within-network connectivity differences distinguished those with TRD from HVs, especially DMN within-network connectivity between the inferior parietal lobule and precuneus in the beta band ( *FDR-corrected p* <.05). Hyperconnectivity within the SN (superior parietal lobule and frontal operculum in the alpha band) and DMN (inferior parietal lobule and lateral prefrontal cortex in the beta band) was associated with number of treatment failures (ps<.05). These findings highlight key brain regions and connectivity patterns, advancing our understanding of neural mechanisms underlying depression and treatment resistance.

## Full Text Availability

The license terms selected by the author(s) for this preprint version do not permit archiving in PMC. The full text is available from the preprint server.

